# Prohibitin 2 deficiency impairs cardiac fatty acid oxidation and causes heart failure

**DOI:** 10.1038/s41419-020-2374-7

**Published:** 2020-03-12

**Authors:** Dechao Wu, Chongshu Jian, Qi Peng, Tingting Hou, Keling Wu, Bizhi Shang, Minglei Zhao, Yanru Wang, Wen Zheng, Qi Ma, Chuan-Yun Li, Heping Cheng, Xianhua Wang, Ling Zhao

**Affiliations:** 1 State Key Laboratory of Membrane Biology, Beijing Key Laboratory of Cardiometabolic Molecular Medicine, Peking-Tsinghua Center for Life Sciences, Institute of Molecular Medicine, Peking University, Beijing, China; 2 State Key Laboratory of Ophthalmology, Zhongshan Ophthalmic Center, Sun Yat-sen University, Guangzhou, China

**Keywords:** Mechanisms of disease, Heart failure

## Abstract

Fatty acids are the most major substrate source for adult cardiac energy generation. Prohibitin 2 (PHB2), a highly conserved protein located in mitochondrial inner membrane, plays key roles in cellular energy metabolic homeostasis. However, its functions in regulating cardiac fatty acid metabolism have remained largely unknown. Our study demonstrates that cardiac-specific knockout of *Phb2* leads to accumulation of lipid droplets and causes heart failure. Mechanistically, ablation of PHB2 impairs cardiac fatty acid oxidation (FAO) through downregulating carnitine palmitoyltransferase1b (CPT1b), a rate-limiting enzyme of cardiac mitochondrial FAO. Moreover, overexpression of CPT1b alleviates impaired FAO in PHB2-deficient cardiomyocytes. Thus, our study provides direct evidence for the link between PHB2 and cardiac fatty acid metabolism. Our study points out that PHB2 is a potential FAO regulator in cardiac mitochondrial inner membrane, as well as the connection between PHB2 and CPT1b and their relationships to cardiac pathology especially to cardiac fatty acid metabolic disorder.

## Introduction

Despite decades of research, heart failure is still a leading cause of morbidity and mortality in modern society^1^. It is a complex clinical syndrome, in which abnormality in cardiac pump function leads to inadequate oxygen supply and perturbed energy metabolism for maintaining normal requirement of cardiac contractility^2^. A variety of mechanisms have been involved in the pathogenesis of heart failure, including metabolic disorder, mitochondrial dysfunction, autophagy, apoptosis, and genetic or epigenetic dysregulation^3–5^. Better understanding the underlying mechanisms of heart failure will provide potential therapeutic targets.

A high rate of ATP production and turnover is required for maintaining normal cardiac functions^2^. As the main source of energy, mitochondrial abnormalities in ATP production may contribute to the pathology of heart failure^6,7^. Prohibitin 2 (PHB2), a highly conserved protein located in mitochondrial inner membrane, plays key roles in cellular energy metabolic homeostasis^8,9^. PHB2 is critical in structural and functional integrity of mitochondria, stability of mitochondrial genome, modulation of mitochondrial dynamics, mitochondrial cristae morphology, respiratory supercomplex formation, mitophagy and mitochondrial quality control^10–15^. However, the function of cardiac PHB2 is less well known, especially in fatty acid metabolic regulation.

Fatty acids are the most major substrate source for adult cardiac ATP generation^16,17^. Fatty acids are mainly fueled via β-oxidation to produce ATP in mitochondria^18^. Severe perturbation in cardiac fatty acid metabolism homeostasis and energy production have been regarded as a consistent feature of heart failure^19,20^. For instance, carnitine palmitoyltransferase-1b (CPT1b), which is the predominant isoform expressed in adult heart, is one of the most important components in controlling fatty acid β-oxidation (FAO)^21,22^. CPT1b deficiency results in impaired cardiac fatty acid oxidation under pressure-overload and exacerbation of cardiac pathology^23^. As mechanisms underlying the regulation of cardiac FAO are complex, signaling pathways of modulating cardiac FAO are still incompletely elucidated.

Loss of *Phb2* in *Caenorhabditis elegans* and mice leads to embryonic lethality^24,25^. Several tissue specific *Phb2* knockout mice have been used to explore its functions in physiological and pathological processes^26–29^. In forebrain-specific PHB2-deficient mice, tau hyper-phosphorylation and neurodegeneration were observed^29^. β-cell-specific knockdown of PHB2 resulted in impaired β-cell function and diabetes^28^. Deletion of PHB2 in podocytes led to the development of glomerulosclerosis due to PHB2’s extra-mitochondria role in the assembly of the slit diaphragm protein-lipid supercomplex^27^. Hepatocyte-specific *Phb2* knockout mice exhibited liver failure and impaired gluconeogenesis^26^. The above studies demonstrate that PHB2 is essential for maintaining normal organ function. Thus, the generation of a cardiac specific *Phb2* knockout mice should greatly help to explore its functions in cardiac energy metabolism in vivo as well.

In our study, we generated cardiac specific *Phb2* knockout mice that exhibited fatty acid oxidation disturbance, mitochondrial dysfunction, and eventually heart failure and lethality, demonstrating that PHB2 is essential for maintaining normal cardiac metabolic functions. Our study also explored whether PHB2 acts as a mitochondrial inner membrane FAO regulator and the relationships between PHB2 and CPT1b in cardiac fatty acid metabolism.

## Results

### Cardiac-specific deletion of *Phb2* leads to dilated cardiomyopathy and heart failure

**Fig. 1:**
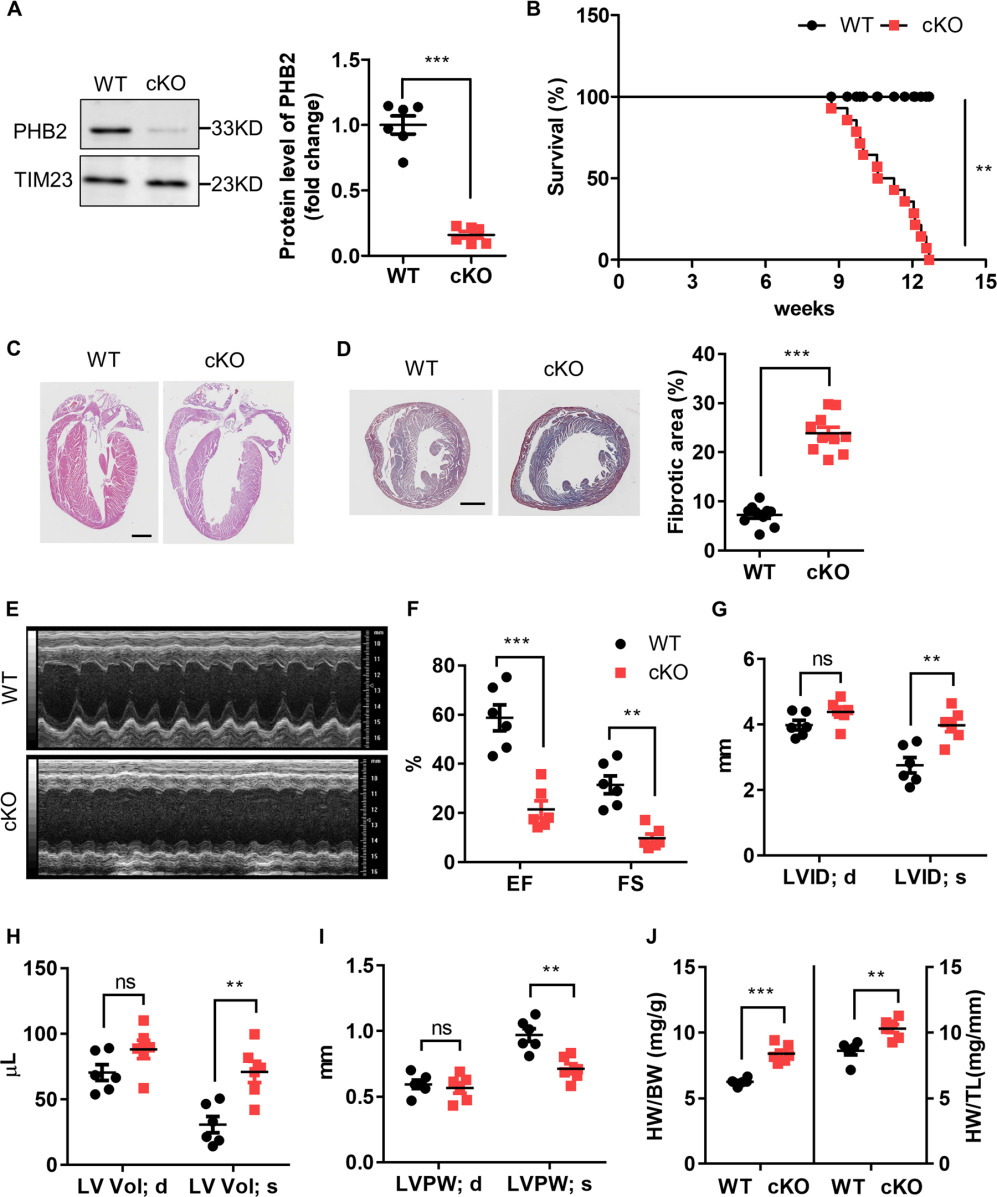
Cardiac-specific knockout of prohibitin 2 (*Phb2*) leads to dilated cardiomyopathy and heart failure. **a** Western blot analysis of PHB2 in the heart tissues from wild type (WT) and *Phb2* cardiac-specific knockout (cKO) mice at 6 weeks of age (left). Anti-TIM23 immunoblot is used as the reference. Statistics for western blot (right). *n* = 6 mice per group. **b** Kaplan-Meier survival curves for WT and *Phb2* cKO mice. *n* = 14 mice per group. Significance was determined by the Kaplan–Meier survival analysis. **p < 0.01 versus WT group. **c** HE staining of gross morphology from WT and *Phb2* cKO mouse hearts at 8 weeks of age. Scale bar: 1 mm. **d** Fibrosis of the heart tissues from 8-week-old WT and *Phb2* cKO mice was assessed by Masson staining (left). Statistics for fibrotic areas (right). *n* = 3 independent experiments per group. Scale bar: 1 mm. **e** Representative echocardiographic images of the left ventricle in WT and *Phb2* cKO mouse hearts at 8 weeks of age. *n* = 6 mice per group. **f** Echocardiographic analysis of EF (ejection fraction) and FS (fractional shortening). **g** Echocardiographic statistics of LVID; d (LV internal diameter at end-diastole) and LVID; s (LV internal diameter at end-systole). **h** Echocardiographic statistics of LV Vol; d (left ventricular volume at end-diastole) and LV Vol; s (left ventricular volume at end-systole). **i** Echocardiographic statistics of LVPW; d (left ventricular posterior wall at end-diastole) and LVPW; s (left ventricular posterior wall at end-systole). **j** The ratio of heart weight (HW) to body weight (BW) and heart weight (HW) to tibia length (TL) from 8-week-old WT and *Phb2* cKO mice. *n* = 6 independent experiments per group. All Data represent mean ± SEM. Significance was determined by two-tailed, unpaired Student’s *t* test. ns: no significance. **p < 0.01, ***p < 0.001 versus WT group.

As whole-body knockout of *Phb2* was embryonic lethal^24,25^, we generated a cardiac-specific *Phb2* knockout (*Phb2* cKO) mouse model wherein exon 4 of *Phb2* was flanked by LoxP sites. The *Phb2*^flox/flox^ mice were cross-bred with Mlc2v-Cre mice to allow cardiac-specific deletion of *Phb2* (Fig. S1). Compared with wild type (WT) littermates, protein expression of cardiac PHB2 was decreased by 80% in *Phb2* cKO mice at 6 weeks of age (Fig. 1a). Cardiac *Phb2* cKO mice exhibited typical phenotypes of dilated cardiomyopathy and heart failure. Compared with WT group, the lifespan of *Phb2* cKO mice was markedly shortened, with sudden death beginning at around 10 weeks of age and a maximal lifespan at 12 weeks of age (Fig. 1b). Ventricular dilation and thinner posterior wall thickness were observed in hematoxylin and eosin (HE) staining of cardiac vertical sections of *Phb2 cKO* mouse hearts at 8 weeks of age (Fig. 1c). Masson staining showed that cardiac ablation of PHB2 induced extensive cardiac fibrosis, and the fibrotic area increased by approximately two-fold compared with WT at 8 weeks of age (Fig. 1d). Moreover, cardiac echocardiography (ECHO) measurements demonstrated cardiac systolic dysfunctions in *Phb2* cKO mice at 8 weeks of age (Fig. 1e), showing both ejection fraction (EF) and fractional shortening (FS) diminished by 70% compared with WT (Fig. 1f). ECHO analysis indicated *Phb2* cKO mouse hearts exhibited enlarged left ventricular inner diameter, increased left ventricular volume and reduced posterior wall thickness during systolic state compared with WT group (Fig. 1g–i). Meanwhile, compared with WT, the heart weight/body weight ratio increased by 34.3%, and the heart weight/tibia length ratio increased by 19.7% in *Phb2* cKO mice at 8 weeks of age (Fig. 1j). In addition, it was found that *Phb2* cKO mice exhibited normal cardiac systolic functions at 4 and 6 weeks of age (Fig. S2A, B, Table S1) and there was no difference in body weight between WT and *Phb2* cKO mice at 8 weeks of age (Fig. S2C). Thus, cardiac specific *Phb2* knockout mouse model is generated and cardiac ablation of *Phb2* results in heart failure shown by shortened lifespan, dilated left ventricle, interstitial fibrosis, and systolic dysfunctions.

### Impaired cardiac energy metabolism and mitochondrial dysfunction are caused by PHB2 ablation

**Fig. 2:**
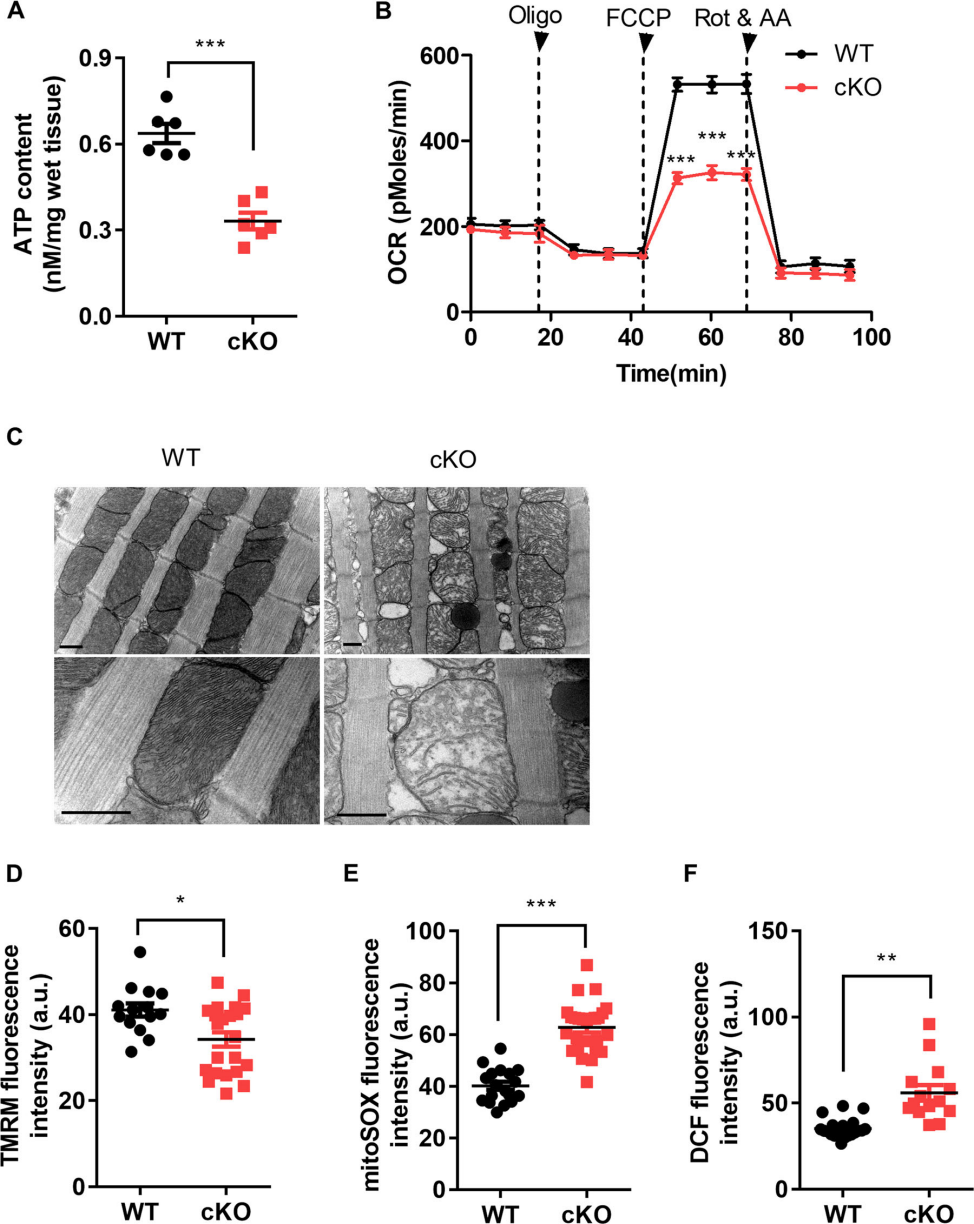
Impaired cardiac energy metabolism and mitochondrial dysfunction are caused by PHB2 ablation. **a** Cardiac ATP levels measured with luciferin assay in 6-week-old WT and *Phb2* cKO mouse hearts. *n* = 6 mice per group. **b** Measurement of oxygen consumption rate (OCR) in isolated cardiomyocytes from 6-week-old WT and *Phb2* cKO mice. Arrowheads indicate the time points of adding Oligo (oligomycin, 1 μm), FCCP (carbonyl cyanide 4-(trifluoromethoxy) phenylhydrazone, 1 μm), Rot (rotenone, 1 μm) and AA (antimycin, 1 μm). *n* = 3 mice per group. **c** Electron micrographs of mitochondrial morphology in left ventricular tissues from 6-week-old WT and *Phb2* cKO mice. Scale bar: 0.5 μm. **d** Statistics of mitochondrial membrane potential measured with TMRM staining in isolated cardiomyocytes from 6-week-old WT and *Phb2* cKO mice. *n* = 80–100 cells from six mice per group. **e** Statistics of mitochondrial ROS levels measured with mitoSOX staining in isolated cardiomyocytes from 6-week-old WT and *Phb2* cKO mice. n = 80–100 cells from six mice per group. **f** Statistics of cytosolic ROS levels measured with DCF staining in isolated cardiomyocytes from 6-week-old WT and *Phb2* cKO mice. *n* = 80–100 cells from six mice per group. All Data represent mean ± SEM. Significance was determined by two-tailed, unpaired Student’s *t* test. *p < 0.05, **p < 0.01, ***p < 0.001 versus WT group.

Cardiac energy metabolism was investigated in 6-week-old *Phb2* cKO mice. Cardiac ATP levels measured with luciferin assay in 6-week-old WT and *Phb2* cKO mouse hearts revealed that PHB2 ablation resulted in lower cellular ATP content (Fig. 2a). The mitochondrial maximal respiration measured by Seahorse assay was prominently suppressed in isolated cardiomyocytes from *Phb2* cKO mice (Fig. 2b). As mitochondrial morphological changes are also related with cardiac dysfunctions^30^, transmission electron microscopy was used to examine cardiac mitochondrial ultrastructure. Compared with WT mice, swollen cardiac mitochondria with few lamellar cristae were observed in *Phb2* cKO mouse hearts (Fig. 2c). Strikingly, lipid droplets (LD) were widely spread around cardiac mitochondria in *Phb2* cKO mouse hearts (Fig. 2c, pyknotic and black circles represented LD), which pointed out that PHB2 might directly regulate cardiac fatty acid metabolism.

We also checked whether PHB2 ablation result in cardiac mitochondrial dysfunction in 6-week-old *Phb2* cKO mice. Using the potential-sensitive fluorescent probe tetramethyl rhodamine methyl ester (TMRM), a significantly decreased mitochondrial membrane potential (ΔΨm) was found in isolated cardiomyocytes from *Phb2* cKO mice (Fig. 2d). Fluorescent probes mitoSOX and DCF were applied to detect mitochondrial and cytosolic ROS, respectively. Both mitochondrial and cytosolic ROS production were markedly elevated in isolated cardiomyocytes from *Phb2* cKO mice compared with WT (Fig. 2e, f). Herein, cardiac mitochondrial dysfunction caused by PHB2 deficiency might partially account for heart failure in *Phb2* cKO mice. Collectively, these results indicated that impaired cardiac energy metabolism and mitochondrial dysfunction are caused by PHB2 ablation.

### Impaired fatty acid oxidation and cardiac lipid accumulation are caused by PHB2 ablation

**Fig. 3:**
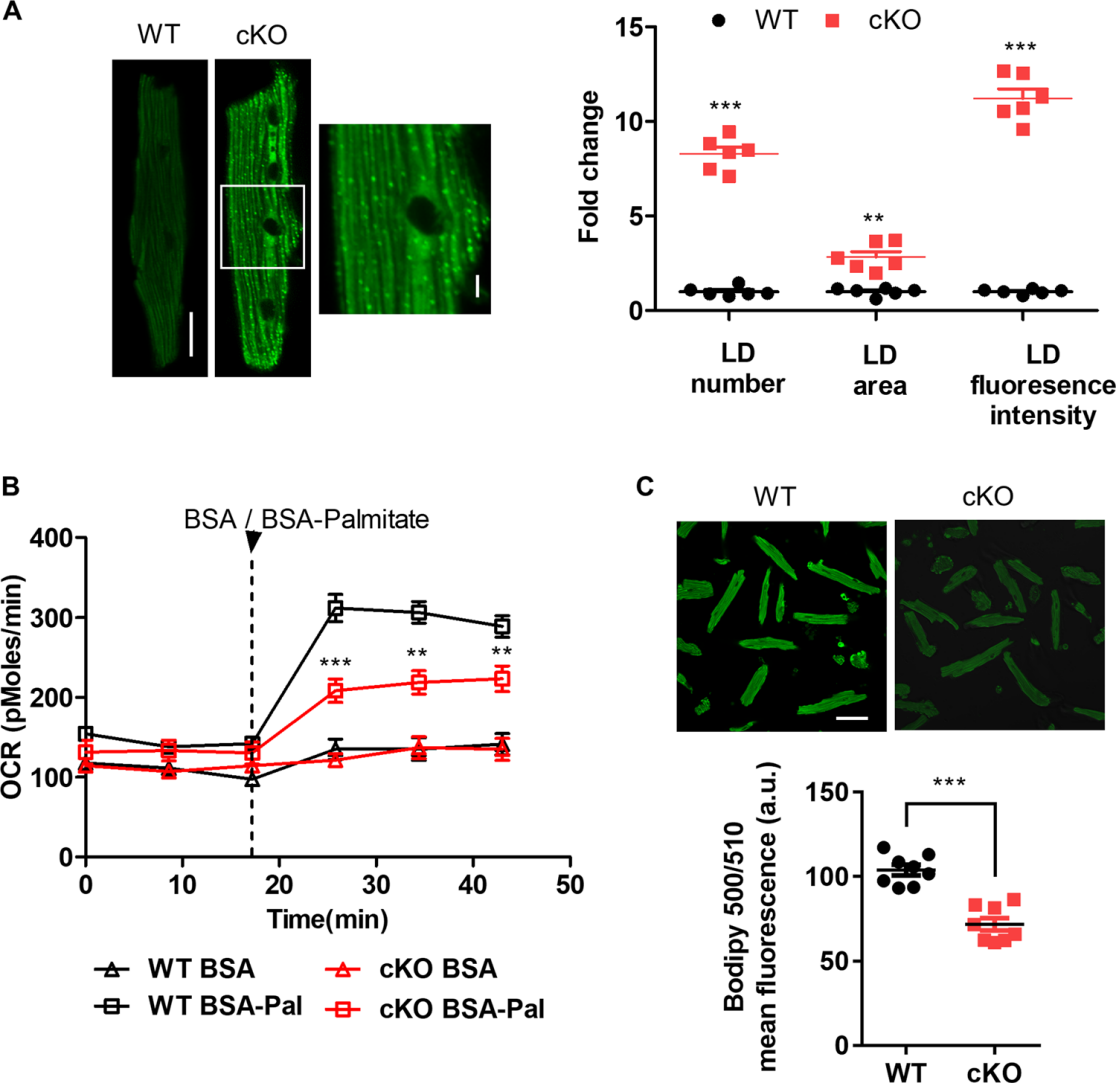
Impaired fatty acid oxidation (FAO) and cardiac lipid accumulation are caused by PHB2 ablation. **a** Representative confocal images of lipid droplets (LD) visualized by BODIPY™ 493/503 in isolated cardiomyocytes from 6-week-old WT and *Phb2* cKO mice (left). Scale bar: 20 μm. The enlargement is the magnification of the boxed inset shown in left panel. Scale bar: 5 μm. Statistics of fold change of LD number, area and fluorescence intensity per cardiomyocyte (right). *n* = 80–100 cells from six mice per group. **b** Measurement of fatty acid oxidation (FAO) in isolated cardiomyocytes from 6-week-old WT and *Phb2* cKO mice. Arrowhead indicates the time points of adding BSA (0.17 mM) or BSA-Pal (Palmitate) (1 mM). *n* = 3 mice per group. **c** Representative confocal images of fatty acid uptake (FAU) visualized by BODIPY™ 500/510 in isolated cardiomyocytes from 6-week-old WT and *Phb2* cKO mice (top). Statistics of FAU levels (bottom). *n* = 80–100 cells from six mice per group. Scale bar: 50 μm. All Data represent mean ± SEM. Significance was determined by two-tailed, unpaired Student’s *t* test. ns: no significance. **p < 0.01, ***p < 0.001 versus WT group.

Unbalanced uptake and oxidation of fatty acids will lead to lipid accumulation in heart tissues, which is also a crucial reason for the occurrence and progression of heart failure^31^. BODIPY 493/503 staining was used for cardiac lipid measurements^32,33^. In our study, LD accumulation was significantly increased, shown by higher LD number, area and higher fluorescence intensity in isolated cardiomyocytes from *Phb2* cKO mice at 6 weeks of age (Fig. 3a). Additionally, Oil Red O staining was performed to examine lipid accumulation in heart tissues and results indicated that much more neutral lipid accumulated in 6-week-old *Phb2* cKO mice compared with WT group (Fig. S3).

We further investigated the underlying correlations between PHB2 and cardiac LD accumulation by testing fatty acid oxidation (FAO) and fatty acid uptake (FAU). It was interesting that both FAO and FAU were markedly reduced in isolated cardiomyocytes from *Phb2* cKO mice. Cardiac FAO was strongly inhibited by PHB2 ablation, which was shown by a much lower FAO of *Phb2* cKO than that of WT with BSA-Palmitate treatment (Fig. 3b). BODIPY 500/510 staining was applied to examine FAU in cardiomyocytes^34^. Results indicated that cardiac FAU was also significantly decreased in *Phb2* cKO mice compared to WT (Fig. 3c). Thus, we suppose that impaired FAO by PHB2 ablation might be a more dominant cause for cardiac lipid accumulation, though decreased FAU might alleviate some cardiac lipid metabolic disorder.

### Proteomic profiles reveal that CPT1b is downstream of PHB2 signaling pathway in cardiac FAO

**Fig. 4:**
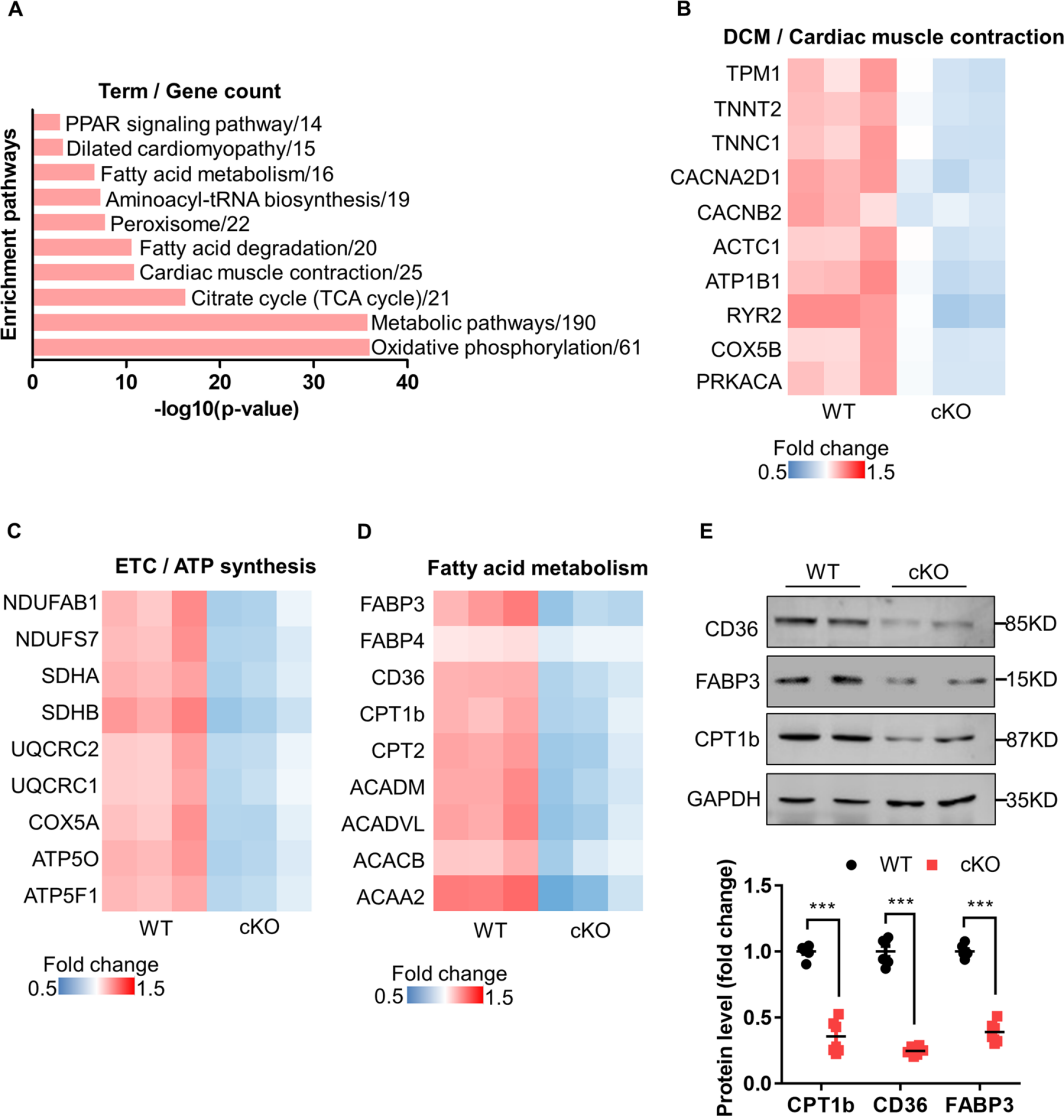
Proteomic profiling of WT and *Phb2* cKO mouse hearts. **a** KEGG pathway analysis of differentially expressed proteins from WT and *Phb2* cKO mouse hearts at 6 weeks of age. **b** Heatmap showing downregulated proteins in the terms “Dilated cardiomyopathy” and “Cardiac muscle contraction” from KEGG pathway analysis in **a**. **c** Heatmap showing downregulated proteins in electron transport chain and ATP synthesis pathways from *Phb2* cKO mouse hearts versus WT. **d** Heatmap of significantly decreased proteins in lipid metabolism from *Phb2* cKO mouse hearts versus WT. **e** Western blot analysis of lipid metabolism proteins (top), including CD36, FABP3 and CPT1b from WT and *Phb2* cKO mouse hearts at 6 weeks of age. Anti-GAPDH immunoblot is used as the reference. Statistics of western blot (bottom), *n* = 3 independent experiments per group. All Data represent mean ± SEM. Significance was determined by two-tailed, unpaired Student’s *t* test. ns: no significance. ***p < 0.001 versus WT group.

To explore the key components downstream of PHB2 deficiency in cardiac fatty acid metabolic homeostasis, we performed quantitative proteomic analysis of WT and *Phb2* cKO mouse hearts at 6 weeks of age. Among 4172 proteins, 931 differentially expressed proteins (fold change ≥ 1.2, *P* value < 0.05) were identified to be associated with distinct biological processes. 418 proteins were significantly upregulated and 513 proteins were significantly downregulated in *Phb2* cKO mouse hearts. KEGG pathways analysis revealed that PHB2 deficiency resulted in a prominent disturbance of oxidative phosphorylation and metabolic pathways (Fig. 4a). Several proteins involved in heart failure were downregulated, which were found with the terms “Dilated cardiomyopathy” and “Cardiac muscle contraction”, such as TPM1 (Tropomyosin 1), TNNT2 (Troponin T2), ACTC1 (Actin, alpha, cardiac muscle 1) (Fig. 4b). As expected, the most enriched pathways were related to mitochondrial function, including ATP biosynthesis, electron transport chain and oxidative phosphorylation (Fig. 4c). Proteomics profiles also revealed that metabolic pathways, especially fatty acid metabolism, was altered by PHB2 deficiency. FABP3 (Fatty acid-binding protein 3), FABP4 (Fatty acid-binding protein 4), CD36 (Cluster of differentiation 36), CPT1b (Carnitine palmitoyltransferase 1b), CPT2 (Carnitine palmitoyltransferase 2), ACADM (Medium-chain specific acyl-CoA dehydrogenase), ACADVL (Very long-chain specific acyl-CoA dehydrogenase), ACACB (Acetyl-CoA carboxylase 2), and ACAA2 (3-ketoacyl-CoA thiolase) were all found to be downregulated in *Phb2* cKO mouse hearts (Fig. 4d). Decreased expression of FABP3, CD36 and CPT1b in *Phb2* cKO mouse hearts was validated by western blot analysis (Fig. 4e). As CPT1b is a rate-limiting enzyme for mitochondrial fatty acid β-oxidation in adult mouse cardiomyocyte^23^, our results indicate CPT1b might be a downstream component of the PHB2 signaling pathway in regulating cardiac FAO.

### Impaired cardiac FAO is caused by PHB2 knockdown in NRVMs

**Fig. 5:**
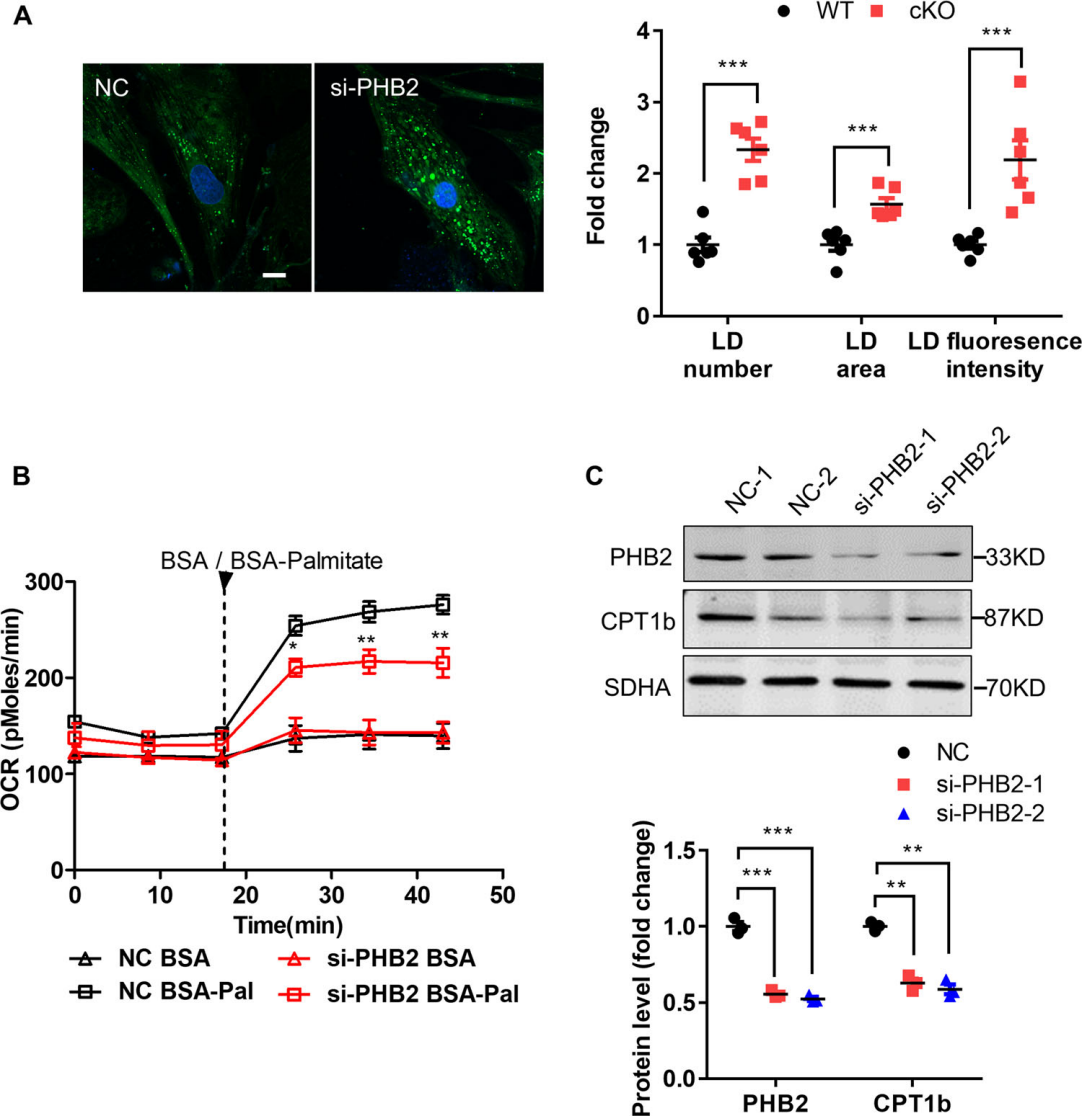
Impaired cardiac FAO in PHB2-deficient neonatal rat ventricular myocytes (NRVMs). **a** Representative confocal images of LD visualized by BODIPY™ 493/503 in NRVMs treated with NC or si-PHB2 (left). Statistics of fold change of LD number, area and fluorescence intensity per NRVM (right). *n* = 100–120 cells from eight independent experiments per group. Scale bar: 10 μm. **b** Measurement of FAO in NRVMs treated with NC or si-PHB2. Arrowhead indicates the time points of adding BSA (0.17 mM) or BSA-palmitate (1 mM). *n* = 3 independent experiments per group. **c** Western blot analysis of PHB2 and CPT1b in NRVMs treated with NC or si-PHB2 (top). Anti-SDHA immunoblot is used as the reference. Two siRNAs were used to target PHB2 (si-PHB2-1 and si-PHB2-2). Statistics of WB (bottom). *n* = 3 independent experiments per group. All Data represent mean ± SEM. Significance was determined by two-tailed, unpaired Student’s *t* test. ns: no significance. *p < 0.05, **p < 0.01, ***p < 0.001 versus NC group. NC: negative control.

We used isolated neonatal rat ventricular myocytes (NRVMs) to explore the underlying mechanisms of PHB2’s regulation of cardiac fatty acid metabolism. In isolated NRVMs, PHB2 knockdown via siRNA (si-PHB2) could induce LD accumulation compared with the negative control (NC) group, reflected by higher LD number, area and higher fluorescence intensity per NRVM (Fig. 5a). FAO of BSA-Palmitate group in si-PHB2 NRVMs was markedly lower than that in NC NRVMs (Fig. 5b), which revealed that PHB2 deficiency significantly inhibited FAO in NRVMs. Decreased expression of CPT1b was detected in si-PHB2 NRVMs (Fig. 5c). As CPT1a is the predominant isoform in fetal cardiomyocytes^21,35^, we also checked its expression in si-PHB2 treated NRVMs. Our results showed there was no significantly difference in CPT1a expression by PHB2 ablation in NRVMs (Fig. S4). In addition, FAU was found to be strongly inhibited in NRVMs treated with si-PHB2 (Fig. S5).

Taken together, in isolated NRVMs, PHB2 knockdown-induced cardiac lipid accumulation is consistent with that in isolated adult ventricular myocytes from *Phb2* cKO mice, which is shown by LD accumulation, reduced FAO and FAU. As CPT1b is downregulated by PHB2 deficiency in heart tissues and NRVMs, we suppose that PHB2 ablation impairs cardiac FAO in part by coordinating with decreased CPT1b.

### Impaired cardiac FAO is alleviated by overexpression of CPT1b in PHB2-deficient NRVMs

Since PHB2 deficiency resulted in impaired cardiac FAO via downregulation of CPT1b, we hypothesized that upregulated expression of CPT1b could alleviate cardiac lipid accumulation.

**Fig. 6:**
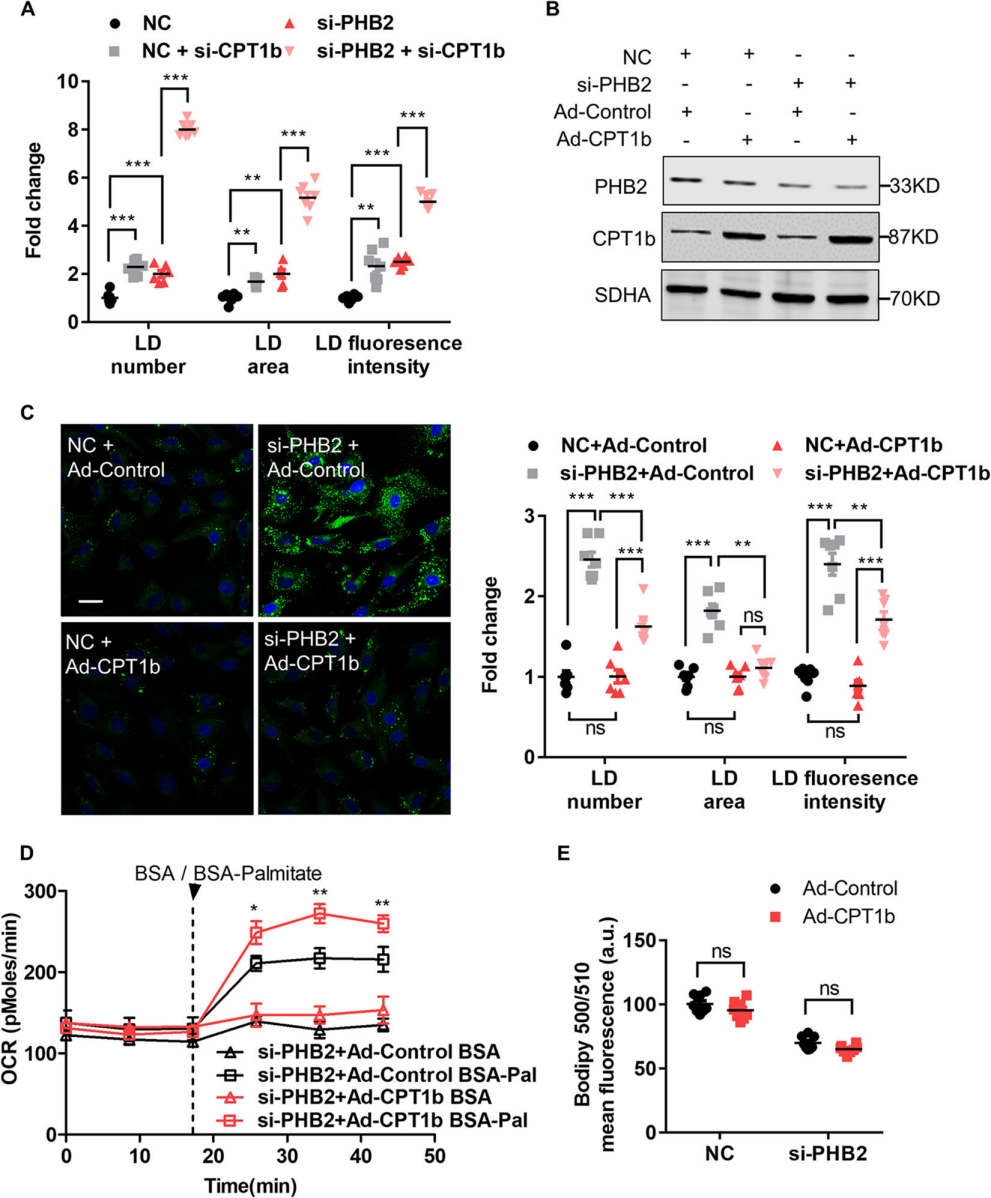
Upregulation of carnitine palmitoyltransferase1b (CPT1b) alleviates cardiac FAO disturbance in PHB2-deficient NRVMs. **a** Measurement of LD visualized by BODIPY™ 493/503 in NRVMs treated with NC, si-PHB2, NC + si-CPT1b, or si-PHB2 + si-CPT1b, respectively. *n* = 100–120 cells from seven independent experiments per group. **b** Western blot analysis of PHB2 and CPT1b in NRVMs treated with NC + Ad-Control, NC + Ad-CPT1b, si-PHB2 + Ad-Control, or si-PHB2 + Ad-CPT1b, respectively. Anti-SDHA immunoblot is used as the reference. **c** Representative confocal images of LD visualized by BODIPY™ 493/503 in NRVMs treated with NC + Ad-Control, NC + Ad-CPT1b, si-PHB2 + Ad-Control, or si-PHB2 + Ad-CPT1b, respectively (left). Statistics of fold change of LD number, area and fluorescence intensity per NRVM (right). *n* = 100–120 cells from six independent experiments per group. Scale bar: 20 μm. **d** Measurement of FAO in NRVMs treated with si-PHB2 + Ad-Control, or si-PHB2 + Ad-CPT1b, respectively. Arrowhead indicates the time point when BSA (0.17 mM) or BSA-palmitate (1 mM) was added. *n* = 3 independent experiments per group. **e** Measurement of FAU visualized by BODIPY™ 500/510 in NRVMs treated with NC + Ad-Control, NC + Ad-CPT1b, si-PHB2 + Ad-Control or si-PHB2 + Ad-CPT1b, respectively. n = 100–120 cells from seven independent experiments per group. All Data represent mean ± SEM. Significance was determined by two-tailed, unpaired Student’s *t* test. ns: no significance. *p < 0.05, **p < 0.01, ***p < 0.001 versus control group.

In si-PHB2 NRVMs, CPT1b siRNA treatment promoted LD accumulation (Fig. 6a). Then we overexpressed CPT1b using adenoviruses in si-PHB2 NRVMs, and the expression of CPT1b and PHB2 was validated by western blot analysis (Fig. 6b). In si-PHB2 NRVMs treated with Ad-CPT1b, LD accumulation was obviously alleviated compared with si-PHB2 and Ad-Control group, reflected in decreased LD number, area and lower fluorescence intensity per NRVM (Fig. 6c). Moreover, FAO of BSA-Palmitate group treated with (si-PHB2 + Ad-CPT1b) was much higher than that treated with (si-PHB2 + Ad-Control) (Fig. 6d). Meanwhile, FAO of BSA-Palmitate group treated with (NC + Ad-CPT1b) was also higher than that treated with (NC + Ad-Control) (Fig. S6). In addition, FAU was not affected by overexpressing CPT1b in NC and si-PHB2 groups (Fig. 6e). Thus, CPT1b alleviated cardiac LD accumulation mainly via upregulating FAO in si-PHB2 NRVMs, rather than affecting FAU. Here, it should be noted that CPT1b overexpression only partially rescued the impairment of FAO caused by PHB2 deficiency, which indicated PHB2 deficiency might play a more dominant or complex role in downregulating FAO.

From the above study, we show that PHB2 plays a critical role in cardiac fatty acid metabolism. Ablation of PHB2 causes heart failure mainly through cardiac FAO disturbance and mitochondrial dysfunction.

## Discussion

### PHB2 deficiency causes heart failure and lethality in mice

Heart failure is one of the leading causes of death worldwide, which is characterized by perturbations in energy production and lack of adequate oxygen supply for maintaining proper heart contractility^36^. As deprivation of cardiac energy plays a major role in heart failure, the modulation of cardiac metabolism has promise as a target for the treatment of heart failure^37,38^.

Prohibitins (PHBs) are highly conserved proteins, which have been involved in multiple physiological and pathological processes. Prohibitins (prohibitin 1 and prohibitin 2) have been shown to play critical roles in aging, cancer, obesity, and mitophagy^8,14,39,40^. However, the function of cardiac PHB2 is still largely unknown. In our study, by generating cardiac specific *Phb2* knockout mice, we demonstrate that PHB2 deficiency significantly impairs cardiac fatty acid oxidation (FAO) and leads to mitochondrial dysfunction, and eventually results in severe heart failure at an earlier age.

### PHB2 is a potential inner mitochondrial membrane FAO regulator

Cardiac-specific *Phb2* knockout mice exhibited severe heart failure shown by dilated left ventricles, interstitial fibrosis, and systolic dysfunction. Accumulation of cardiac lipid droplets (LD) and mitochondrial metabolic dysfunction were also detected in *Phb2* cKO hearts. Thus, our study indicated that PHB2 is critical for maintaining the homeostasis of cardiac lipid metabolism.

As we know, the preferentially utilized substrate is fatty acid in healthy cardiac muscle. In a fasting state, myocardial fatty acid oxidation provides almost 70% of cardiac ATP^41^. Impaired FAO has been shown to play a key role in multiple mechanisms of heart failure^41,42^. In other organs, PHBs have been shown to be involved in lipid metabolism. For example, in Mito-Ob mice, PHBs have been shown to be involved in metabolic regulation of adipose tissue homeostasis^39^. Another study discovered loss of PHB2/DNAJC19 complexes affects cardiolipin acylation and leads to the accumulation of cardiolipin species^43^. However, the functions of PHB2 in cardiac fatty acid metabolism have not yet been reported.

In our study, PHB2 was identified as a potential FAO regulator required for homeostasis of cardiac fatty acid metabolism. In isolated cardiomyocytes from *Phb2* cKO mice and si-PHB2 NRVMs, large numbers of LD significantly accumulated, shown by higher LD number, area and higher fluorescence intensity. More importantly, PHB2 deficiency resulted in decreased CPT1b, and upregulated expression of CPT1b partially alleviated cardiac lipid accumulation caused by PHB2 deficiency. In summary, cardiac PHB2 deficiency-induced mitochondrial dysfunction resulted in fatty acid metabolic disorder through downregulating CPT1b, which played a major role in the course of heart failure in *Phb2* cKO mice. Here, different from increased body weight in obesity or diabetes mouse models with lipotoxic cardiomyopathy, there was no difference in body weight between WT and *Phb2* cKO mice. Therefore, it suggested that PHB2 may play a unique role in modulating cardiac fatty acid metabolism.

### Studying PHB2 signaling will contribute to a novel treatment strategy for heart failure

The pathogenesis of heart failure is very complex. As we described above, to elucidate the mechanisms for modulating homeostasis of cardiac metabolism will greatly help to develop promising treatments for heart failure. In our study, we found that PHB2 deficiency in the heart impaired cardiac FAO and reduced ATP level, and that these defects in *Phb2* cKO hearts occurred at 6 weeks of age, prior to the occurrence of heart failure which was evident at 8 weeks of age. Therefore, our study provided the evidence for causal relationships between FAO impairment and heart failure. Meanwhile, our study revealed the connection between PHB2 and CPT1b in cardiac fatty acid oxidation and upregulation of CPT1b that could partially alleviate lipid accumulation caused by PHB2 deficiency. Thus, our study indicated that CPT1b might be a potential therapeutic target for heart failure. However, it is controversial with current therapy for acute heart failure using CPT1b inhibitor^16,41^. The ablation of cardiac PHB2 exhibits a more severe heart failure at an early age (50% mortality at day 70); however, haploinsufficiency of CPT1b mice showed heart failure only under a severe pressure-overload condition. Thus, decreased cardiac FAO might partially account for heart failure. In our study, mitochondrial dysfunction shown by profound ultrastructural alteration in cristae morphology, elevated ROS, reduced mitochondrial membrane potential and decreased ATP content were found in *Phb2* cKO mouse hearts. We supposed that ablation of PHB2 might trigger additional stress to intensify cardiac dysfunction.

In addition, accumulating evidences show that autophagy is critical in metabolic homeostasis maintenance, protein quality control and mitochondrial network remodeling, which offers autophagy as an emerging target for treating cardiorenal metabolic disease^4^. Here, as PHB2 is an inner mitochondrial membrane mitophagy receptor^14^ and autophagy is reported to be involved in disturbed lipid metabolism^44–46^, the roles of PHB2 on cardiac autophagy in heart failure or other cardiac pathologies should be further investigated in future studies.

Taken together, our findings provide direct evidence that PHB2 plays an essential role in cardiac fatty acid metabolism homeostasis and also demonstrate that CPT1b is downstream of PHB2 signaling pathway. *Phb2* cKO mice exhibited severe heart failure and died at an average age 10 weeks, with cardiac lipid accumulation and mitochondrial dysfunction. Thus, our study points to the distinct possibility that PHB2 is an inner mitochondrial membrane FAO regulator. Further studies on PHB2’s control of mitochondrial metabolic homeostasis will contribute to develop or optimize therapeutic approaches to heart failure.

## Materials and methods

### Animal care

All animal experiments were carried out according to the rules of the American Association for the Accreditation of Laboratory Animal Care International and the Guide for the Care and Use of Laboratory Animals published by the US National Institutes of Health (NIH Publication No. 85-23, revised 1996). All procedures were approved by the Animal Care Committee of Peking University accredited by AAALAC International.

### Reagents

Dulbecco’s modified Eagle’s medium (DMEM), Lipofectamine RNAiMax, penicillin and streptomycin were from Invitrogen (Carlsbad, CA). Fetal bovine serum (FBS) was from Gibco (Carlsbad, CA). MitoSOX, DCF, TMRM, BODIPY™ 500/510, BODIPY™ 493/503 were from Molecular Probes (Eugene, OR). Mycoplasma contamination was detected using Mycoplasma Detection Kit (Beyotime, China).

### Strategy of generating cardiac-specific *Phb2* knockout mice

The cardiac-specific knockout mice were generated as previously described^47^. Floxed *Phb2* mice were obtained from the Nanjing Biomedical Research Institute of Nanjing University. Exon4 of the *Phb2* gene was inserted into two flanking LoxP sites. Homozygous *Phb2*-floxed mice (*Phb2*^flox/flox^) were obtained by inbreeding of the *Phb2*^flox/+^ mice, which were generated from C57BL/6 J mice by the CRISPR/Cas9 system. Briefly, *Phb2*^flox/flox^ mice were bred with Mlc2v-Cre mice in which Cre recombinase expression was controlled by the myosin light chain 2 v promoter to generate double heterozygous Mlc2v-Cre and *Phb2* floxed mice (Mlc2v-Cre^+^ and *Phb2*^flox/+^). The mice were then backcrossed with homozygous *Phb2*^flox/flox^ mice to generate Mlc2v-Cre^+^ and *Phb2*^flox/flox^ as cardiac-specific *Phb2* knockout mice, Mlc2v-Cre^-^ and *Phb2*^flox/flox^ mice as littermate controls.

Mice were genotyped by PCR using mouse tail DNA, and flox primers (forward, TGCTCTGGAGAAAGTGCCCC; reverse, CACACACCACAAACAGCAACAG) and Cre primers (forward, GCGGTCTGGCAGTAAAAACTATC; reverse, GTGAAACAGCATTGCTGTCACTT), respectively. PCR program: 95 °C, 10 min; 95 °C, 30 s, 52 °C, 30 s, 72 °C, 45 s (35 cycles); 72 °C, 5 min; 4 °C, hold.

### Histology and immunohistochemistry

The hearts were perfused with 2.5% glutaraldehyde and 4% paraformaldehyde at 8 weeks of age. Then the hearts were fixed in 4% paraformaldehyde overnight, bisected transversely at the mid-ventricular level, embedded in paraffin and cut into 4 mm sections for staining with hematoxylin and eosin (HE). Additionally, heart sections were stained with Masson’s trichrome for fibrosis analysis. Oil Red O staining on frozen sections was performed to examine lipid accumulation in hearts. Eight regions were randomly assigned from the full-size crosscut heart for quantification of Masson staining, each consisting of a rectangle of 515 × 434 μm^2^. Image J was used for fibrosis areas quantification.

### Echocardiography

Male mice were randomly chosen to be anesthetized with isoflurane, and performed transthoracic echocardiography using a VEVO-2100 Imaging System (Visual Sonics) at 4, 6, 8 weeks of age, respectively. The group allocations for echocardiography were reviewed blindly by the investigator. Fractional shortening (FS) and ejection fraction (EF) were used to assess systolic function. Left ventricular posterior wall thickness at end-diastole and end-systole (LVPW; d and LVPW; s), left ventricular internal diameter at end-diastole and end-systole (LVID; d and LVID; s), left ventricular volume at end-diastole and end-systole (LV Vol; d and LV Vol; s) were obtained from the M-mode image.

### Isolation of adult mouse cardiomyocytes

Single ventricular myocytes were isolated from the hearts of randomly chosen wild type, *Phb2* cardiac-specific knockout mice at 6 weeks of age, as previously described^48–50^. Freshly-isolated cardiomyocytes were plated on culture dishes covered with laminin (Sigma) for at least 1 h and then the attached cells were in DMEM medium (Invitrogen, Carksbad, California) along with 10% FBS (Gibco), 5 mM BDM (Sigma), and 1% Insulin-Transferrin-Selenium (ITS) Supplement (Invitrogen) until use.

### Isolation and culture of neonatal rat ventricular myocytes

Ventricular myocytes were isolated from 1-day-old Sprague-Dawley rats, as previously described^51^. Neonatal rat ventricular myocytes (NRVMs) were plated at 1.5 × 10^5^ cells/cm^2^ in DMEM supplemented with 10% FBS and 1% penicillin and streptomycin in the presence of 0.1 mM 5-bromo-2-deoxyuridine (Sigma). Adenovirus infection or siRNA transfection was implemented after 24 h quiescence in serum-free DMEM following 48–72 h culture in DMEM containing 10% FBS.

### Cell culture, siRNA transfection and adenovirus infection

For RNA interference, the negative control was used as control for all siRNA experiments. 100 nM siRNA were transiently transfected into the NRVMs with RNAi Max according to the manufacturers’ instructions. The siRNAs used in this study were listed (Table S2).

For adenovirus infection, NRVMs were infected with adenovirus carrying the C terminal Myc-tagged CPT1b gene at a multiplicity of infection (m.o.i.) of 20. Confocal imaging and western blot analysis were performed 72 h after siRNA transfection and 48 h after virus infection.

### Confocal microscopy and image processing

Zeiss LSM710 inverted confocal microscope with a 40 × 1.3 NA oil-immersion objective was used for acquiring images. All experiments were carried out with Tyrode’s solution (137 mM NaCl, 5.4 mM KCl, 1.2 mM MgCl2, 1.2 mM NaH2PO4, 1.8 mM CaCl2, 10 mM glucose, and 20 mM HEPES, pH 7.35, adjusted with NaOH) at room temperature (22–26 °C).

For measuring mitochondrial ROS, mitoSOX (5 μM) was loaded into isolated cardiomyocytes in Tyrode’s solution at 37 °C for 10 min and washed three times before imaging. Fluorescence of mitoSOX was taken by excitation at 514 nm and emission collection at >500 nm.

For measuring cytosolic ROS levels, DCF (5 μM) was loaded into isolated cardiomyocytes in Tyrode’s solution at 37 °C for 20 min and washed three times before imaging. Fluorescence of DCF was taken by excitation at 488 nm and emission collection at >500 nm.

For measuring mitochondrial membrane potential, TMRM (100 nM) was loaded into isolated cardiomyocytes in Tyrode’s solution at 37 °C for 10 min. Fluorescence of TMRM was taken by excitation at 543 nm and emission collection at >560 nm.

For detecting fatty acids uptake, BODIPY™ 500/510 (20 μM) was loaded into isolated cardiomyocytes for 2 min in Tyrode’s solution at 37 °C and washed three times before imaging. BODIPY™ 500/510 fluorescence were both taken by excitation at 488 nm and emission collection at 500–550 nm.

For visualizing LD, BODIPY™ 493/503 (20 μM) was loaded into isolated cardiomyocytes for 10 min in Tyrode’s solution at 37 °C and washed three times before imaging. BODIPY™ 493/503 fluorescence were both taken by excitation at 488 nm and emission collection at 500–550 nm.

Confocal images were analyzed using Interactive Data Language (IDL, Research Systems) software and customer-devised programs.

### Western blot

Heart tissues or isolated cardiomyocytes were homogenized and lysed in lysis buffer (30 mM HEPES, 100 mM NaCl, 0.5% Nonidet P (NP)-40, protease inhibitors mixture, pH 7.6) on ice for 10 min, and the lysates were centrifuged at 13,000 rpm for 20 min. Cell lysates (50 μg/line) were separated by 12% SDS-PAGE and transferred to PVDF 0.2 mm membranes. Membranes were blotted with 5% dry, non-fat milk prepared in Tris-buffer saline-plus 0.1% Tween-20 (TBST) at room temperature for one hour and incubated with primary antibodies diluted in 5% milk overnight at 4 °C. Blots were visualized using secondary antibodies conjugated with IRDye (LI-COR) by an Odyssey imaging system (LI-COR).

The following antibodies were used for western blotting: PHB2 (1:1000, CST, 14085), SDHA (1:2000, Abcam, ab14715), CPT1b (1:1000, Abcam, ab134988), TIM23 (1:2000, BD Transduction Laboratories™, 611222), GAPDH (1:2000, ABclonal, AC002), CD36 (1:500, Thermo Fisher, PA1-16813), FABP3 (1:1000, Proteintech, 10676-1-AP), CPT1a (1:1000, Proteintech, 15184-1-AP).

### Transmission electron microscopy

Fresh excised hearts from 6-week-old mice were perfused by 1% glutaraldehyde and 4% paraformaldehyde for 30 min. After dissecting into 1–2 mm^3^ blocks, samples were immediately fixed with 2.5% glutaraldehyde and 4% paraformaldehyde, then post-fixed with 1% osmium tetroxide/1.5% potassium ferrocyanide. Following several washes in distilled water, samples were stained with 2% aqueous uranyl acetate for 2 h at room temperature. After rinsing several times in distilled water, the specimens were dehydrated through a graded alcohol series and subsequently embedded in Spurr’s resin (SPI supplies, PA, USA). Ultra-thin sections (70 nm) were cut with a diamond knife using an ultramicrotome (UC7, Leica Microsystem), and collected on copper grids with a single slot. After counterstaining with uranyl acetate and lead citrate, sections were examined using an electron microscopy (Tecnai G2 20 TWIN, FEI) at 120 kV.

### Measurement of mitochondrial respiratory function

The mitochondrial respiration was measured using XF24 Extracellular Flux Analyzer (Seahorse Bioscience, North Billerica, MA) according to the manufacturer’s instructions. Briefly, isolated cardiomyocytes were cultured in XF24 cell-culture microplates at 10^5^ cells/well in DMEM supplemented with 10% FBS. After adherence, the culture medium was changed to assay medium (Sigma D5030) supplemented with 2 mM GlutaMAX (Gibco), 2.5 mM sodium pyruvate, and 25 mM glucose (pH7.4 at 37 °C). Then cardiomyocytes were incubated for 1 h at 37 °C prior to measurement. Bioenergetic analyses were performed in an XF24 Extracellular Flux Analyzer with oligomycin (1 μM), FCCP (1 μM), rotenone (1 μM), and antimycin A (AA, 1 μM) injected sequentially. After each drug injection, the OCR was measured three times. Basal OCR refers to the respiration rate measured prior to the addition of oligomycin. Maximal OCR was calculated by subtracting the OCR in the presence of rotenone and AA from that in the presence of FCCP.

### Measurement of mitochondrial fatty acid oxidation

Isolated cardiomyocytes were cultured in XF24 cell-culture microplates at 300 cells/well in DMEM supplemented with 10% FBS. After adherence, the culture medium was changed to Krebs-Henseleit Buffer (KHB component: NaCl 111 mM, KCl 4.7 mM, MgSO4 2 mM, Na2HPO4 1.2 mM, Glucose 2.5 mM, Carnitine 0.5 mM), and cardiomyocytes were incubated for 1 h at 37 °C prior to measurement. 1 mM BSA-Palmitate solution and 0.17 mM BSA solution were prepared according to XF BSA-Palmitate FAO Reagent instructions. BSA-Palmitate or BSA was injected at the time points. Mitochondrial fatty acid oxidation was calculated by subtracting the OCR in the presence of BSA from that in the presence of BSA-Palmitate.

### ATP measurement

The ATP concentration in heart was measured with the Enlighten ATP assay system using luciferase and luciferin (Promega). Briefly, the hearts were excised and lysed with Tris-phenol. The myocardial ATP content was measured with the luciferase assay according to manufacturer’s instructions.

### Quantitative proteomic analysis by tandem mass tag (TMT) technology

For each sample (cKO VS WT), 20 μg of proteins were mixed with 5× loading buffer and boiled for 5 min. The proteins were then separated on 12.5% SDS-PAGE gel (constant current 14 mA, 90 min) and protein bands were visualized by Coomassie Blue R-250 staining for quality control. After that, 200 μg of proteins from each sample were prepared essentially as previously described with filter-aided sample preparation (FASP Digestion)^52^.

Using TMT reagents according to the manufacturer’s instructions, 100 μg of the peptide mixture from each sample was labeled (Thermo Scientific, Massachusetts, USA). A Pierce high pH reverse-phase fractionation kit (Thermo Scientific) was used to fractionate TMT-labeled digested samples into 15 fractions by an increasing acetonitrile step-gradient elution carried according to the instructions.

Liquid chromatography-mass spectrometry/MS (LC-MS/MS) analysis was performed on a Q Exactive mass spectrometer (Thermo Scientific) that was coupled to Easy nLC for 60 min. The mass spectrometer was operated in positive ion mode. Tandem mass spectrometry (MS/MS) spectra were searched using MASCOT engine (Matrix Science, London, UK; version 2.2) embedded into Proteome Discoverer 1.4.

The differentially expressed proteins were identified by fold change values of greater than ±1.2 and *p* < 0.05 (from Mann–Whitney U-tests). The KEGG pathway enrichment analysis for both upregulated and downregulated genes were performed using Database for Annotation, Visualization, and Integrated Discovery^53^ with a P-value cutoff of 0.05 under the Benjamini test.

### Statistics

The data are presented as mean ± SEM, and two-tailed Student’s *t* test was applied to determine statistical significance. *P* < 0.05 was considered statistically significant. The log-rank test was used for survival curves. We did not use any specific test to estimate statistical power. Sample size was chosen based on the previous experience obtained from the study of mouse models or biological measurements. The number of replications is listed in each figure legend.

## Supplementary information

Supplementary Tables Figures legends

Supplemental table 1

Supplemental table 2

Supplemental Figure 1

Supplemental Figure 2

Supplemental Figure 3

Supplemental Figure 4

Supplemental Figure 5

Supplemental Figure 6
