# Supplementary material for: Prohibitin 2 deficiency impairs cardiac fatty acid oxidation and causes heart failure
Source: Cell Death Dis. 2020 Mar 12;11(3):181. doi: 10.1038/s41419-020-2374-7 (PMC7067801; doi:10.1038/s41419-020-2374-7)
Supplement: Supplementary file 1 — Supplementary Tables Figures legends [file 41419_2020_2374_MOESM1_ESM.docx]

**Supplementary Tables and Figures Legends**

**Supplemental Table 1.** *In vivo* cardiac function of WT and *Phb2* cKO mice at different ages. EF (ejection fraction) and FS (fractional shortening). LVID; d (LV internal diameter at end-diastole) and LVID; s (LV internal diameter at end-systole). LVPW; d (left ventricular posterior wall at end-diastole) and LVPW; s (left ventricular posterior wall at end-systole). LV Vol; d (left ventricular volume at end-diastole) and LV Vol; s (left ventricular volume at end-systole). n = 6 mice per group. All Data represent mean ± SEM. Significance was determined by two-tailed, unpaired Student’s t test. ***p* < 0.01, ****p* < 0.001 versus the corresponding WT group.

**Supplemental Table 2.** The sequence of NC, si-PHB2 and si-CPT1b used in this study.

**Supplemental Figure 1.** Schematic of gene targeting strategy. LoxP sites flank exon 4 of *Phb2* gene by the CRISPR/Cas9 system. *Phb2*^flox/flox^ mice were crossed with Mlc2v-Cre to allow cardiac-specific deletion of *Phb2*. HR: homologous recombination.

**Supplemental Figure 2.**

**(A)** Echocardiographic analysis of EF (ejection fraction) and FS (fractional shortening) in the left ventricle from WT and *Phb2* cKO mouse hearts at 4 weeks of age. n = 6 mice per group.

**(B)** Echocardiographic analysis of EF and FS in the left ventricle from WT and *Phb2* cKO mouse hearts at 6 weeks of age. n = 6 mice per group.

**(C)** Statistics of body weight from WT and *Phb2* cKO mice at 8 weeks of age. n = 12 mice per group.

All Data represent mean ± SEM. Significance was determined by two-tailed, unpaired Student’s t test. ns: no significance. ns: no significance versus WT group.

**Supplemental Figure 3.** Oil red O staining of heart tissues from WT and *Phb2* cKO mice at 6 weeks of age. Scale bar: 500 μm (left), 50 μm (right). Black arrows indicated areas of lipid accumulation in heart tissues.

**Supplemental Figure 4.** Western blot analysis of CPT1a in NRVMs treated with NC or si-PHB2 (top). Anti-SDHA immunoblot is used as the reference. Statistics of WB (bottom). n = 3 independent experiments per group.

All Data represent mean ± SEM. Significance was determined by two-tailed, unpaired Student’s t test. ns: no significance versus NC group. NC: negative control.

**Supplemental Figure 5.** Representative confocal images of FAU visualized by BODIPY™ 500/510 in NRVMs treated with NC or si-PHB2 (top). Scale bar: 20 μm. Statistics of FAU (bottom). n = 100 ~ 120 cells from 12 independent experiments per group.

All Data represent mean ± SEM. Significance was determined by two-tailed, unpaired Student’s t test. ns: no significance. ****p* < 0.001 versus NC group. NC: negative control.

**Supplemental Figure 6.** Measurement of FAO in NRVMs treated with NC + Ad-Control, or NC + Ad-CPT1b, respectively. Arrowhead indicates the time point when BSA (0.17 mM) or BSA-palmitate (1 mM) was added. n = 3 independent experiments per group.

All Data represent mean ± SEM. Significance was determined by two-tailed, unpaired Student’s t test. **p* < 0.05, ***p* < 0.01 versus control group.
