# Supplementary material for: Prohibitin 2 deficiency impairs cardiac fatty acid oxidation and causes heart failure
Source: Cell Death Dis. 2020 Mar 12;11(3):181. doi: 10.1038/s41419-020-2374-7 (PMC7067801; doi:10.1038/s41419-020-2374-7)
Supplement: Supplementary file 2 — Supplemental table 1 [file 41419_2020_2374_MOESM2_ESM.docx]

**Supplemental Table 1.**

|  | **4 weeks** | | **6 weeks** | | **8 weeks** | |
| --- | --- | --- | --- | --- | --- | --- |
|  | WT | cKO | WT | cKO | WT | cKO |
| EF (%) | 49.45±6.29 | 56.85±7.97 | 52.25±4.89 | 51.90±3.35 | 58.73±12.88 | 21.48±8.55*** |
| FS (%) | 28.03±4.14 | 32.10±4.92 | 25.46±2.93 | 25.93±2.03 | 31.45±8.93 | 9.75±4.33** |
| LVID; d (mm) | 3.85±0.08 | 3.37±0.07 | 3.71±0.13 | 3.21±0.08 | 3.98±0.36 | 4.38±0.38 |
| LVID; s (mm) | 2.90±0.13 | 2.23±0.15 | 2.86±0.26 | 2.43±0.04 | 2.75±0.58 | 3.98±0.49** |
| LVPW; d (mm) | 0.45±0.06 | 0.55±0.15 | 0.61±0.14 | 0.71±0.09 | 0.59±0.08 | 0.57±0.10 |
| LVPW; s (mm) | 0.62±0.06 | 0.78±0.05 | 0.81±0.12 | 0.86±0.05 | 0.97±0.12 | 0.71±0.09** |
| LV; d (µL) | 64.08±3.23 | 57.71±1.82 | 58.61±4.95 | 51.29±2.44 | 70.46±14.79 | 88.08±17.07 |
| LV; s (µL) | 32.30±3.38 | 33.21±1.43 | 31.56±6.97 | 30.77±0.78 | 30.71±14.79 | 70.98±19.82** |
