# Supplementary material for: Prohibitin 2 deficiency impairs cardiac fatty acid oxidation and causes heart failure
Source: Cell Death Dis. 2020 Mar 12;11(3):181. doi: 10.1038/s41419-020-2374-7 (PMC7067801; doi:10.1038/s41419-020-2374-7)
Supplement: Supplementary file 3 — Supplemental table 2 [file 41419_2020_2374_MOESM3_ESM.docx]

| siRNA name | sense sequence (5'-3') | antisense sequence (5'-3') |
| --- | --- | --- |
| NC | UUCUCCGAACGUGUCACGUTT | ACGUGACACGUUCGGAGAATT |
| si-PHB2-1 | GGACGAUGUAGCUAUCACATT | UGUGAUAGCUACAUCGUCCTT |
| si-PHB2-2 | GCGUACAACAGGACACAAUTT | AUUGUGUCCUGUUGUACGCTT |
| si-CPT1b-1 | GCCUUAUUCGAAUCAAGAATT | UUCUUGAUUCGAAUAAGGCTT |
| si-CPT1b-2 | CCAAAGAAUUCCAGGACAATT | UUGUCCUGGAAUUCUUUGGTT |

**Supplemental Table 2**
