# Supplementary figures and images for: Prohibitin 2 deficiency impairs cardiac fatty acid oxidation and causes heart failure
Source: Cell Death Dis. 2020 Mar 12;11(3):181. doi: 10.1038/s41419-020-2374-7 (PMC7067801; doi:10.1038/s41419-020-2374-7)

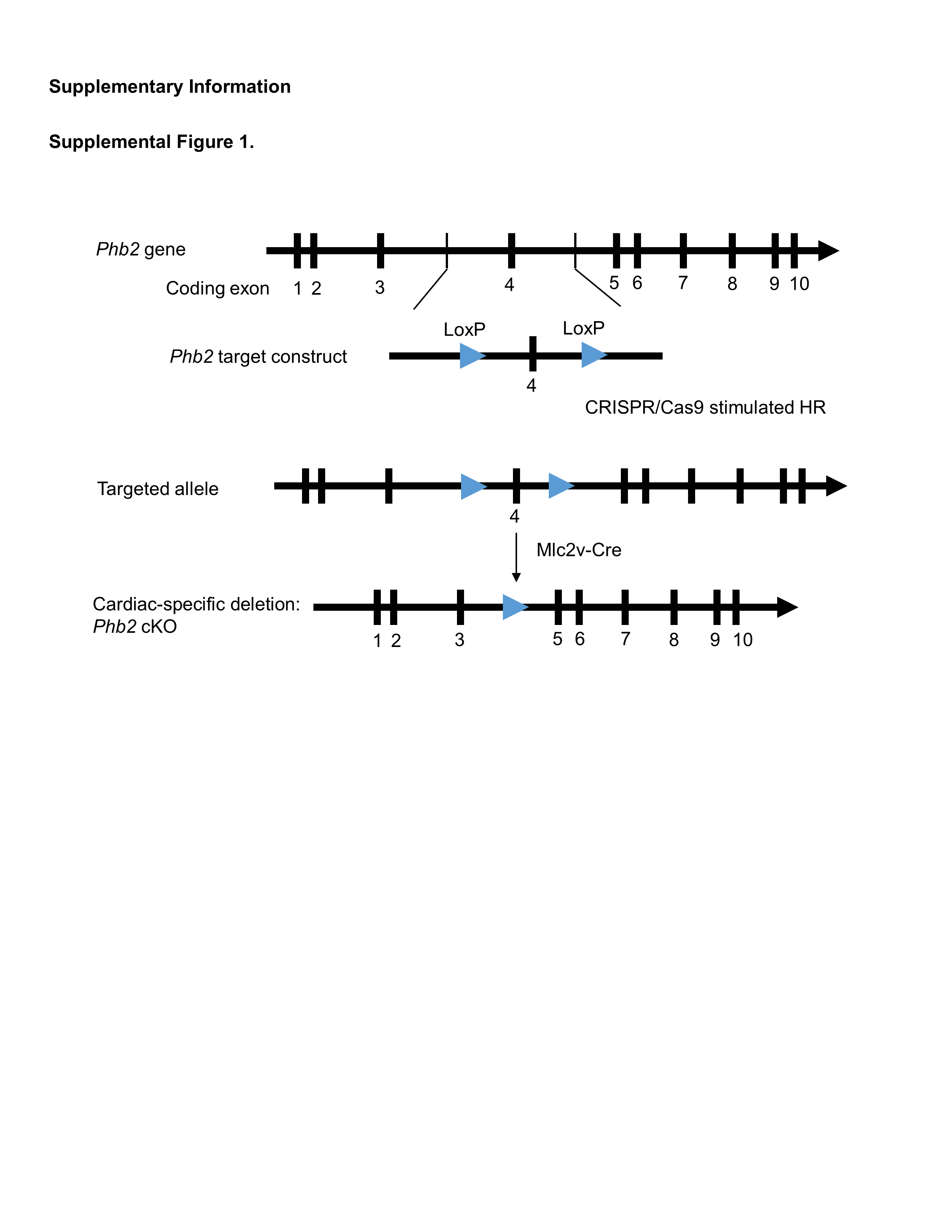

Supplement: Supplementary file 4 — Supplemental Figure 1 [file 41419_2020_2374_MOESM4_ESM.tif]

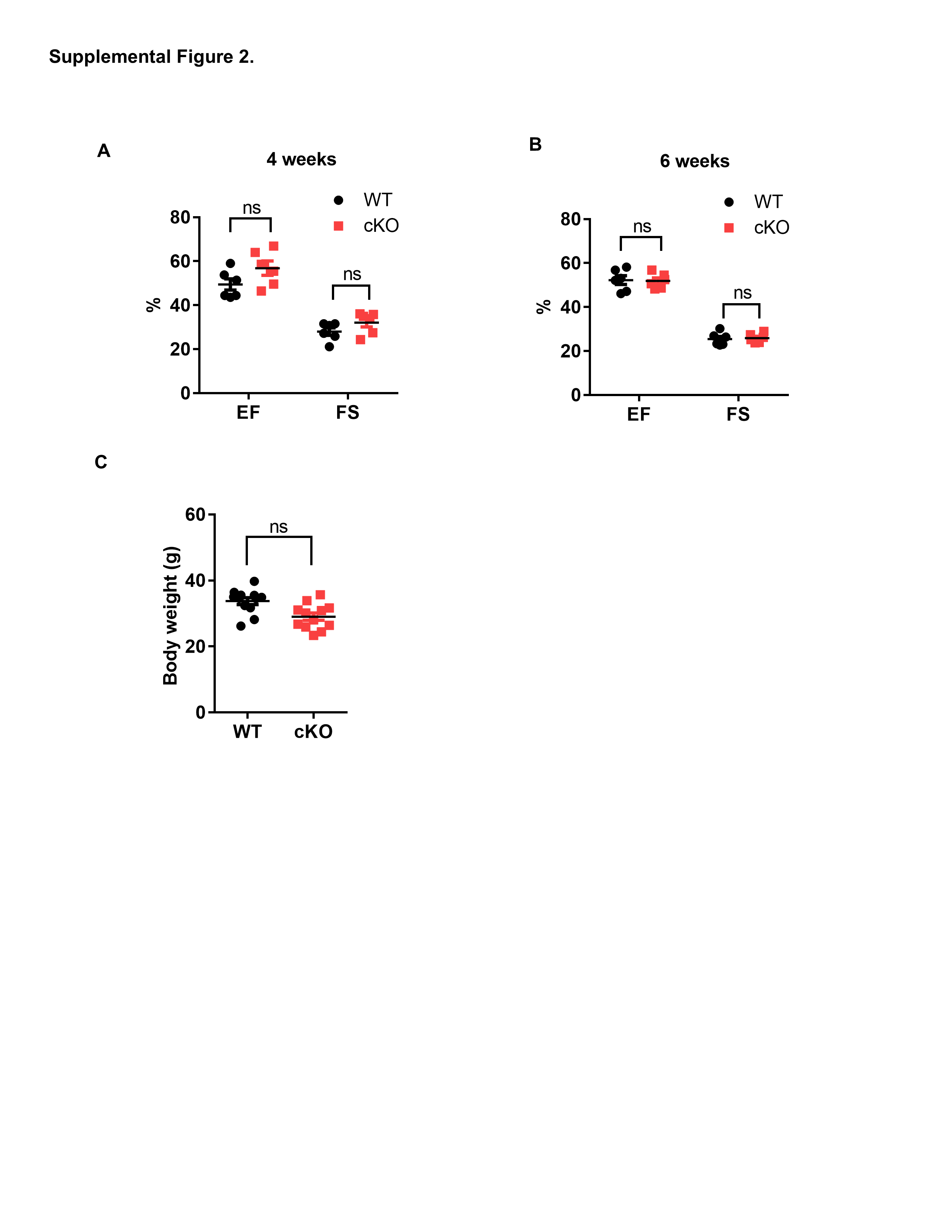

Supplement: Supplementary file 5 — Supplemental Figure 2 [file 41419_2020_2374_MOESM5_ESM.tif]

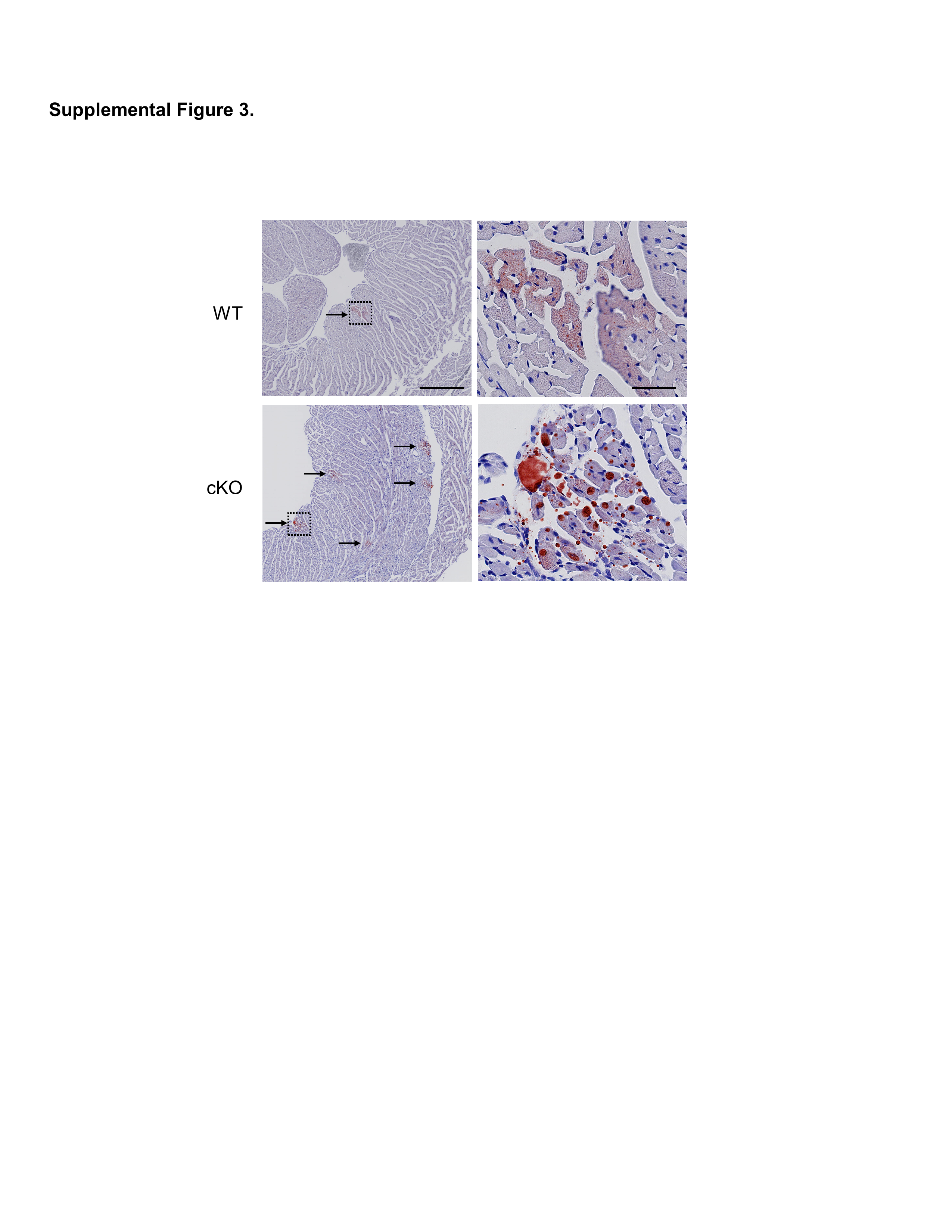

Supplement: Supplementary file 6 — Supplemental Figure 3 [file 41419_2020_2374_MOESM6_ESM.tif]

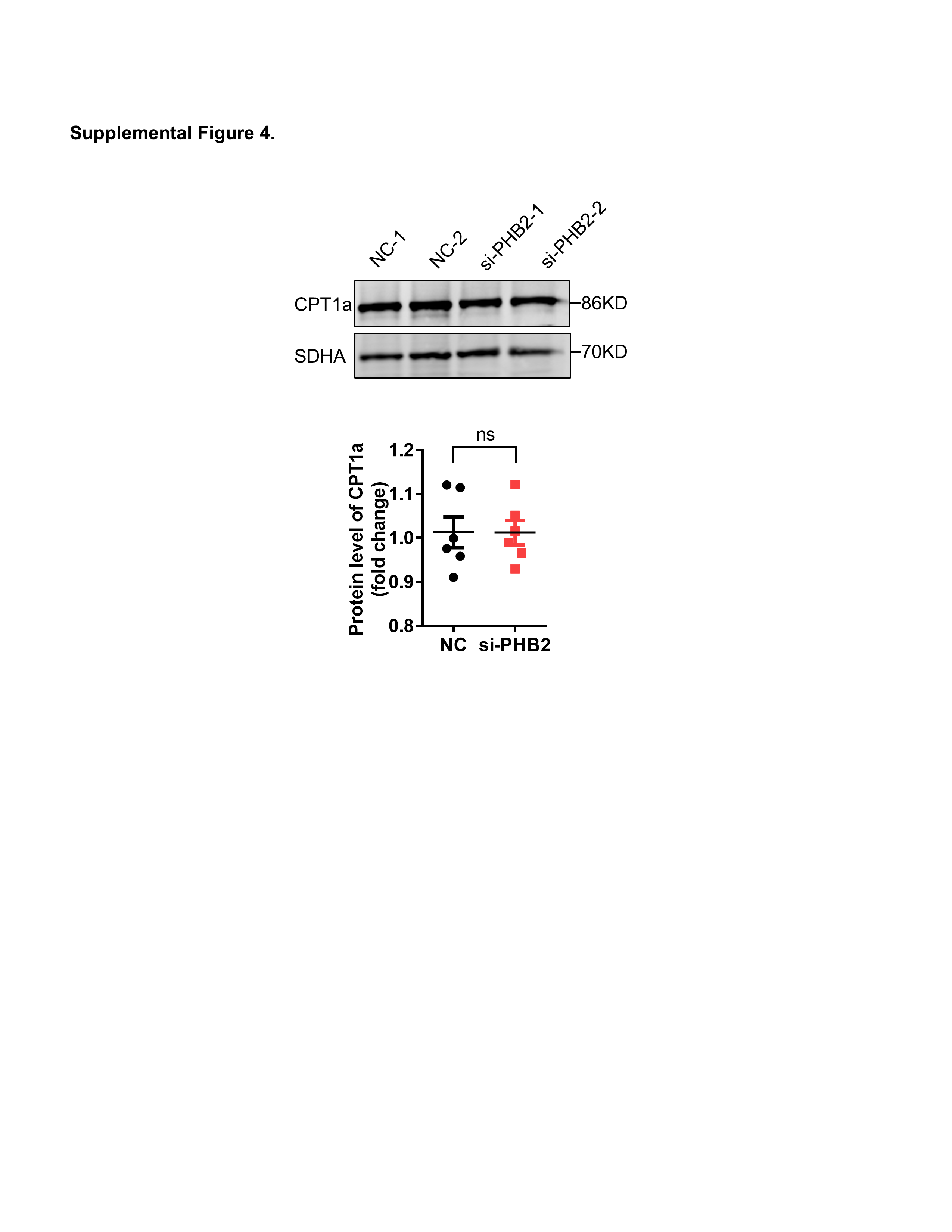

Supplement: Supplementary file 7 — Supplemental Figure 4 [file 41419_2020_2374_MOESM7_ESM.tif]

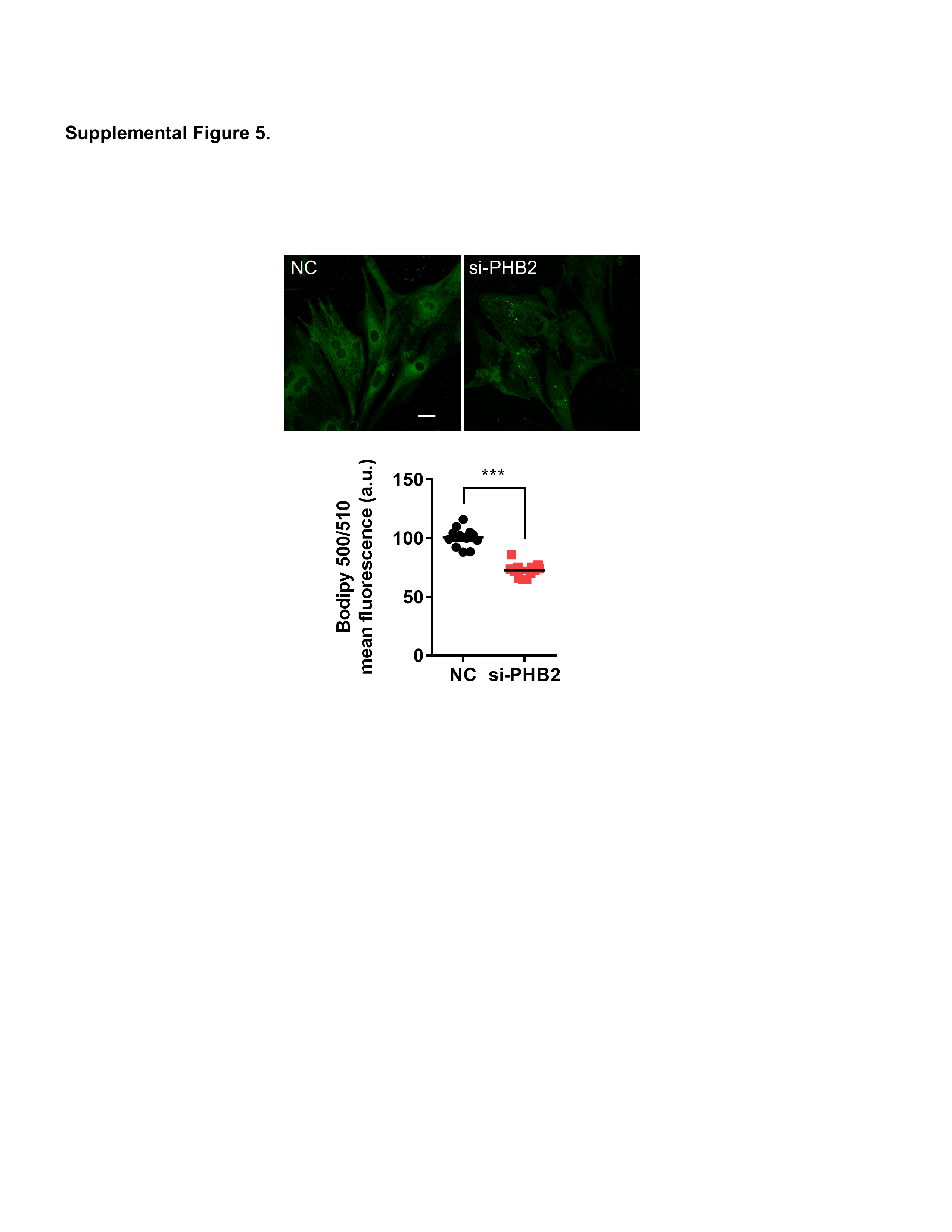

Supplement: Supplementary file 8 — Supplemental Figure 5 [file 41419_2020_2374_MOESM8_ESM.tif]

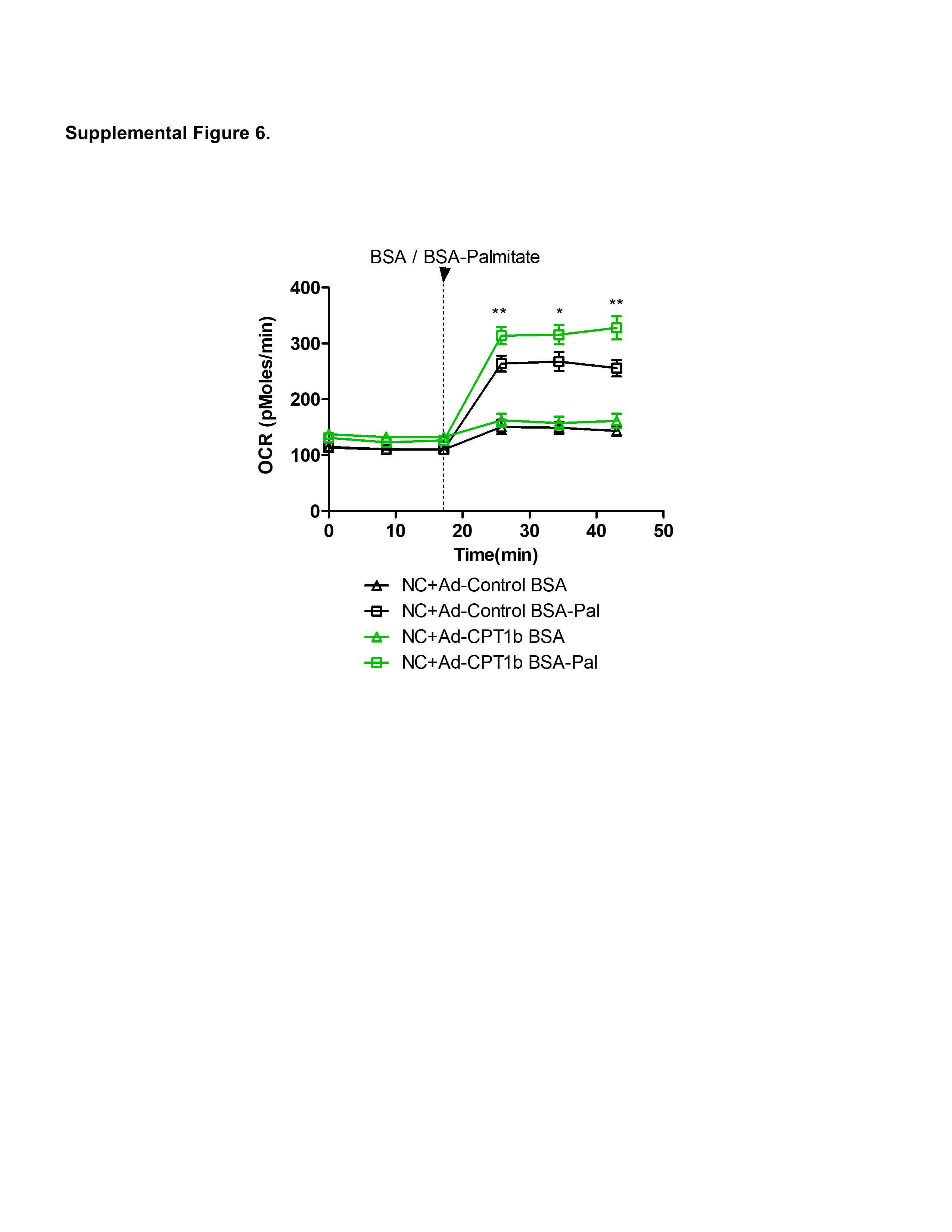

Supplement: Supplementary file 9 — Supplemental Figure 6 [file 41419_2020_2374_MOESM9_ESM.tif]
